# Systems biology approach to identify transcriptome reprogramming and candidate microRNA targets during the progression of polycystic kidney disease

**DOI:** 10.1186/1752-0509-5-56

**Published:** 2011-04-25

**Authors:** Priyanka Pandey, Shan Qin, Jacqueline Ho, Jing Zhou, Jordan A Kreidberg

**Affiliations:** 1 Department of Medicine, Children's Hospital Boston; Department of Pediatrics, Harvard Medical School, Boston, MA, 02115, USA; 2 Current address: Division of Nephrology, Department of Pediatrics, University of Pittsburg School of Medicine, Pittsburg, PA, 15224, USA; 3 Department of Medicine, Brigham and Women's Hospital and Department of Medicine, Harvard Medical School. Boston, MA, 02115, USA; 4 Harvard Stem Cell Institute, Cambridge, MA, 02138, USA

## Abstract

**Background:**

Autosomal dominant polycystic kidney disease (ADPKD) is characterized by cyst formation throughout the kidney parenchyma. It is caused by mutations in either of two genes, *PKD1 *and *PKD2*. Mice that lack functional *Pkd1 *(*Pkd1*^*-/-*^), develop rapidly progressive cystic disease during embryogenesis, and serve as a model to study human ADPKD. Genome wide transcriptome reprogramming and the possible roles of micro-RNAs (miRNAs) that affect the initiation and progression of cyst formation in the *Pkd1*^*-/- *^have yet to be studied. miRNAs are small, regulatory non-coding RNAs, implicated in a wide spectrum of biological processes. Their expression levels are altered in several diseases including kidney cancer, diabetic nephropathy and PKD.

**Results:**

We examined the molecular pathways that modulate renal cyst formation and growth in the *Pkd1*^*-/- *^model by performing global gene-expression profiling in embryonic kidneys at days 14.5 and 17.5. Gene Ontology and gene set enrichment analysis were used to identify overrepresented signaling pathways in *Pkd1*^*-/- *^kidneys. We found dysregulation of developmental, metabolic, and signaling pathways (e.g. Wnt, calcium, TGF-β and MAPK) in *Pkd1*^*-/- *^kidneys. Using a comparative transcriptomics approach, we determined similarities and differences with human ADPKD: ~50% overlap at the pathway level among the mis-regulated pathways was observed. By using computational approaches (TargetScan, miRanda, microT and miRDB), we then predicted miRNAs that were suggested to target the differentially expressed mRNAs. Differential expressions of 9 candidate miRNAs, miRs-10a, -30a-5p, -96, -126-5p, -182, -200a, -204, -429 and -488, and 16 genes were confirmed by qPCR. In addition, 14 candidate miRNA:mRNA reciprocal interactions were predicted. Several of the highly regulated genes and pathways were predicted as targets of miRNAs.

**Conclusions:**

We have described global transcriptional reprogramming during the progression of PKD in the *Pkd1*^*-/- *^model. We propose a model for the cascade of signaling events involved in cyst formation and growth. Our results suggest that several miRNAs may be involved in regulating signaling pathways in ADPKD. We further describe novel putative miRNA:mRNA signatures in ADPKD, which will provide additional insights into the pathogenesis of this common genetic disease in humans.

## Background

Autosomal dominant polycystic kidney disease (ADPKD) is characterized by fluid-filled cysts that are thought to result from abnormal cell proliferation and deregulated apoptosis, increased secretion of fluids into the tubular lumen, irregular cell-matrix interactions, and defective cellular polarity [1,2]. Thus, normal parenchyma is replaced by a cystic epithelium and fibrotic tissue [3]. Genetic mutations in *PKD1 *(encoding polycystin 1; PC-1) are responsible for majority of cases of ADPKD, the remainder are due to loss of *PKD2 *(encoding polycystin 2; PC-2). Loss of PC-1 or PC2 expression results in disruption of intracellular Ca^2+ ^levels, which may lead to abnormal proliferation of tubule epithelial cells [4-7]. Additionally, the involvement of PC-1 in various pathways related to proliferation, such as G-protein signaling, Wnt signaling, AP-1, and cell cycle arrest has been reported [8-12]. However, the manner in which these diverse pathways are integrated into cellular circuitry and regulated during progression of ADPKD is not well studied.

MicroRNAs (miRNAs) are small endogenous non-protein encoding RNAs that post-transcriptionally modulate gene expression by binding to the 3'UTR of target mRNAs [13]. They are involved in many biological processes including cell differentiation, cell proliferation, cell mobility and apoptosis [14] and are associated with many diseases including cancer, hypertension, diabetes, and kidney dysfunction [15]. For example, Kato et al reported a role for miR-192 in diabetic nephropathy [16]. Also, over-expression of the miR-17-92 cluster may play a role in renal cell carcinoma [17]. Further, the importance of miRNAs that are expressed in kidney is supported by mouse knockout studies [18-21]. For example, eliminating Dicer, a key enzyme in miRNA biogenesis, from podocytes, a cell type required for the formation of the size exclusion barrier in the glomerulus, results in progressive loss of podocyte function [20,21]. Some studies have also suggested a role for miRNAs in ADPKD [22-24].

The *Pkd1*^*-/- *^mouse model develops cystic disease caused by mutation of the same gene responsible for the majority of human ADPKD, and provides a system to study the pathogenesis of ADPKD [25]. However, a systematic, large-scale study, elucidating global changes in gene expression during disease progression in the *Pkd1*^*-/- *^mouse model has yet to be reported. Using the *Pkd1*^*-/- *^model to study the pathogenesis of ADPKD offers the ability to compare gene expression in pre-diseased and diseased kidneys. In the current investigation, we (1) use the *Pkd1*^*-/- *^model to explore the transcriptional changes that occur in ADPKD on a whole genome scale, (2) undertake a comparative transcriptomics approach to determine similarities and differences with human ADPKD, and (3) investigate whether these changes might be related to changes in miRNA expression. We systemically predicted the possible miRNAs that may be associated with the changes in mRNA expression levels during disease progression that were determined by gene expression microarray analysis. We predicted miRNAs that could target signaling pathways in ADPKD. Our results suggest that several miRNAs may be involved in regulating the genetic switches in ADPKD. We further describe several miRNAs and putative miRNA-mRNA signatures, which were previously not reported in ADPKD.

## Methods

### Animal Model

Kidneys from wild type (WT; *Pkd1*^*+/+*^) and *Pkd1*-null, designated as *Pkd1*^*-/-*^, mutant littermates were investigated at the embryonic ages 14 (E14.5) and 17 (E17.5). The kidneys were fixed in 4% paraformaldehyde overnight, paraffin-embedded, and stained with hematoxylin and eosin. The sections were visualized with a Nikon Eclipse 80i microscope and photographed with a Qimaging Retiga 2000R Fast 1394 camera using NIS-Elements Basic Research 2.34 software (Micro Video Instruments, Avon, MA).

### Total RNA extraction and Microarrays

Total RNA was extracted from embryonic kidneys at, E14.5 and E17.5, using the Qiagen miRNeasy Mini kit (Qiagen, Valencia, CA). Three pairs of kidneys at E14.5 and E17.5 were processed. Each pair was analyzed separately. The integrity and purity of the mRNA samples were assessed prior to hybridization using Bioanalyser 2100 with mRNA Nanochips (Agilent Technologies). RNA hybridization was performed, and gene expression profiles were determined using Illumina Mouse Sentrix 6 version 2 Beadchips. The data extraction was performed by using an Illumina Bead Studio V3.1.3.0 software with the output being raw, non-normalized bead summary values.

The raw data matrix extracted from Beadstudio was uploaded in Bioconductor R version 2.9.1 for downstream analysis. The raw data were read using the beadarray package available through the Bioconductor project. The raw intensities were background adjusted by the Subtract method. The log2 summarized data were quantile normalized. The limma package [26] with empirical Bayes method was used to assess the differentially expressed genes.

The data discussed in this publication have been deposited in NCBI's Gene Expression Omnibus (GEO) and are accessible through GEO Series accession number GSE24352 (http://www.ncbi.nlm.nih.gov/geo/query/acc.cgi?acc=GSE24352).

### Gene set enrichment analysis and Gene Ontology enrichment analysis

**Figure 1:**
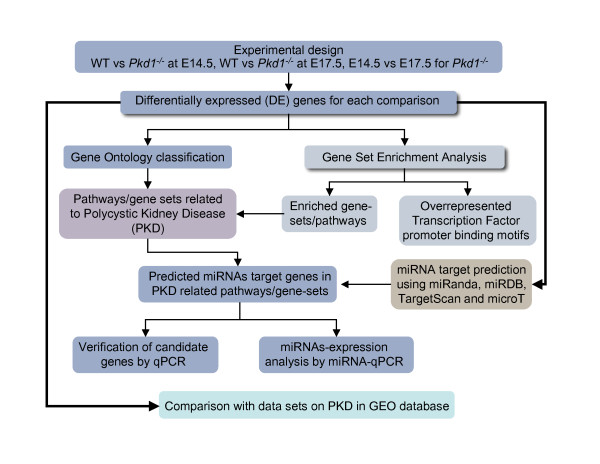
**Schematic representation of combinatorial approach identifying genes, molecular pathways and target miRNAs in ADPKD**. mRNA expression profilings at E14.5 and E17.5 were analysed by limma package in Bioconductor R. Differentially expressed (DE) genes were obtained for comparisons- Mutant vs. WT at E14.5, at E17.5 and E14.5 vs E17.5 for mutants. Gene Ontology (GO) was used to compare the dominant biological processes in mutant and WT at each time point. Gene set enrichment analysis (GSEA) was performed to identify gene pathways associated with PKD progression and renal cyst growth; and to search for overrepresented TF promoter binding motifs among DE genes. Target miRNAs for DE genes at each comparison were predicted using TargetScan, miRanda, miRDB and microT and results were overlapped using Perl scripts. A subset of DE genes associated with PKD (as revealed from GO and GSEA analysis) and targeted by miRNAs (revealed by target prediction tools), was selected for verification by qPCR. miRNAs predicted to target DE genes by atleast two tools, were selected for verification by qPCR. Additionally, comparison of DE genes with other data sets on ADPKD were performed to derive set of pathways core to ADPKD.

Gene set enrichment analysis (GSEA) (http://www.broad.mit.edu/gsea/) was used to identify potential gene pathways and key transcription factors (TFs) that may modulate cystogenesis (Figure 1). The GSEA C2 database (curated gene sets from BioCarta, KEGG, Signaling Pathway database, Signaling Gateway, Signal Transduction Knowledge Environment, Human Protein Reference database, GenMAPP, Sigma-Aldrich Pathways, Gene Arrays, BioScience Corp., Human Cancer Genome Anatomy Consortium database) that includes well-studied metabolic and signaling pathways and published microarray data sets was used for pathway analysis. The GSEA C3 database containing shared and evolutionarily conserved TF binding motifs defined by the TRANSFAC database was used for TF analysis. The description of each gene set can be found on the GSEA Molecular Signatures Database website: http://www.broad.mit.edu/gsea/msigdb/index.jsp Enrichment of Gene Ontology (GO) categories (Figure 1) in the differentially expressed genes was determined using Bioconductor's GOstats package [27] for both up- and down-regulated genes.

### Comparison with other datasets on ADPKD

Two datasets on ADPKD with mutation in *Pkd1, Pkd1*^*L3/L3 *^[28] and human ADPKD [29] were obtained from the GEO database (http://www.ncbi.nlm.nih.gov/geo/; [30]). Raw data were log2 summarized and quantile normalized. The limma package [26] with empirical Bayes method was used to assess the differentially expressed genes between mutants and controls. These were compared with the differentially expressed genes obtained in comparisons using custom written Perl scripts.

### miRNA prediction

As shown in Figure 1, a combinatorial strategy was used where target miRNAs were predicted for the differentially expressed genes using four algorithms, TargetScan (http://www.targetscan.org/; [31]), miRanda (http://www.microrna.org/; [32]), miRDB (http://mirdb.org/miRDB/; [33]) and microT (http://diana.cslab.ece.ntua.gr/microT/; [34]). To identify the miRNAs commonly predicted by two or more algorithms, results were intersected (Additional file 1) using custom written Perl scripts (Additional file 2).

### Quantitative real-time PCR

Total RNA was extracted from *Pkd1*^*-/- *^and WT kidneys. Three independent biological replicates (different from those used for microarrays) were used and all reactions were run in duplicates for all the expression analysis of genes and miRNAs. For all the quantitative real-time-PCR (qPCR) assays, the ABI PRISM 7300 Sequence Detection System was used. The comparative CT method was used to obtain relative quantitation of genes and miRNAs as per the manufacturer's protocol (Qiagen). For gene expression analysis, cDNA was made using Superscript III reverse transcriptase (Invitrogen). qPCRs were performed, using Power SYBR^® ^Green PCR Master Mix (Invitrogen). Primer sets for P2rx7, Cpeb3, Hdac9, Sox6, Ltbp1, Calcr, Pitx2, Fgfr3, Fgf10, Adam22, Ddx3y, F2rl2, Grap2, Edil3, Mysm1, Alg6 and Alg8 are available in Additional file 3. 18S rRNA was used as the endogenous control.

miRNA reverse transcription was performed using the TaqMan microRNA Reverse Transcription Kit and miRNA-specific primers (Applied Biosystems, Foster City, CA). Real-time TaqMan miRNA-assays were used to quantify miRNA expression using the TaqMan Master Mix and validated primer/probe sets (Applied Biosystems). miRNA levels were normalized to snoRNA-202. Cycling conditions for TaqMan PCR consisted of an initial incubation at 50°C for 2 min and 95°C for 15 sec and 60°C for 1 min.

## Results

### Pkd1^-/- ^mice model and design of experiment

**Figure 2:**
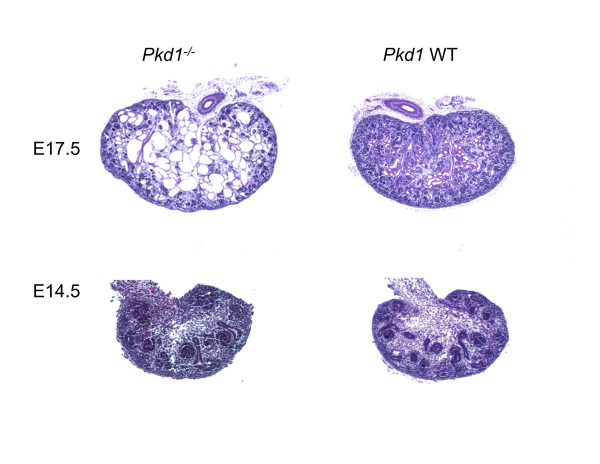
**Pathology of *Pkd1*^*-/- *^mouse model at embryonic ages: E14.5 and E17.5**. At E14.5 the mutant and WT kidneys are similar. Kidney development in *Pkd1*^*-/- *^mutants appears to proceed normally until E15.5. By E17.5, the kidneys of *Pkd1*^*-/- *^mutants are filled with many large numbers of renal cysts. The rate of cyst development indicates aggressive disease in *Pkd1*^*-/- *^mutants. Hematoxylin/Eosin stained; Scale bar 20x.

**Figure 3:**
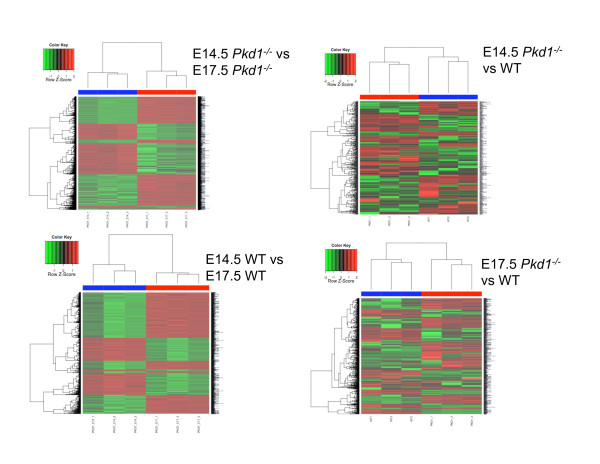
**Differential expression of gene in PKD and control animals**. Heatmap was produced using simultaneous clustering of rows and columns of the data matrix using complete linkage algorithm and a euclidean distance metric. Prior to clustering, values were transformed to zero (row-wise) mean and unit (row-wise) variance. The gene clustering tree is shown on the left and the sample clustering tree is shown on the top. The samples are clustering broadly into two groups, wild type (WT) and PKD. The color scale shown at the right illustrates the relative expression level of the indicated gene across all samples: red denotes expression > 0 and green denotes an expression < 0. Genes shown here are from mRNA microarrays.

**Figure 4:**
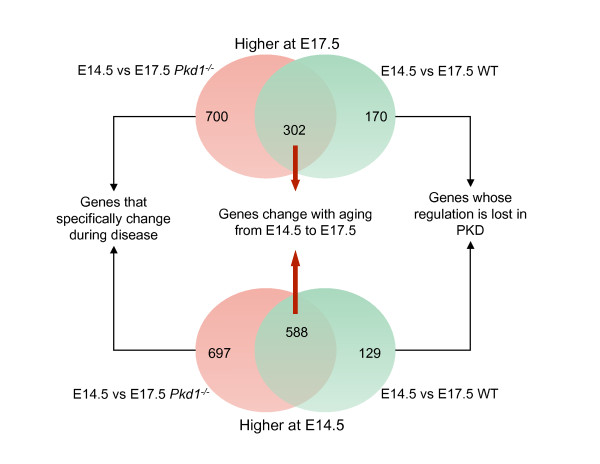
**Genes specific to aging and transition under healthy and diseased conditions**. Venn diagram shows genes that are specific to transition under healthy and diseased conditions as well as genes changing with aging from the embryonic age 14 to 17. The red circles show genes changing only in mutants and green circles show genes changing only in wild-types. The overlapped from both the comparisons yielded genes changing with aging and common to both mutants and wild-types.

To study the detailed changes in molecular profiles during the progression of ADPKD, we generated and compared gene expression profiles of *Pkd1*^*-/- *^embryonic kidneys and age-matched WT kidneys at two stages, E14.5 and E17.5 (Figure 1; Additional file 4). *Pkd1*^*-/- *^embryos develop rapidly progressive kidney cysts during embryogenesis, with null mutant kidneys showing no cysts at E14.5 and marked cystic changes by E17.5 (Figure 2). Unsupervised hierarchical clustering was able to discriminate WT and mutant kidney samples at both time points (Figure 3). At the same time, the cluster analysis was also able to distinguish changes between E14.5 and E17.5 kidneys (Figure 3). Table 1 shows a summary of number of differentially expressed genes and pathways for all of the comparisons. Genes showing a greater than 2-fold difference in expression between WT and mutant kidneys at E14.5 as well as at E17.5 (empirical Bayes moderated t-statistic, unequal variance, uncorrected p-value ≤0.05), were considered to be differentially expressed whereas in the comparisons of WT kidneys at E14.5 vs E17.5 and *Pkd1*^*-/- *^kidneys at E14.5 vs E17.5, genes with ≥ 2-fold difference in expression at p-value ≤0.05 (corrected for multiple testing by Benjamini-Hochberg method) were considered as differentially expressed. The expression of 454 genes was significantly changed between WT and mutant kidneys at E14.5 (Table 1, Additional file 5) whereas 884 genes were significantly changed at E17.5 between WT and mutant kidneys (Table 1, Additional file 6). The comparison of E14.5 and E17.5 WT kidneys yielded 1189 differentially expressed genes (Additional file 7) whereas 2287 genes were differentially expressed in comparison of *Pkd1*^*-/- *^kidneys at E14.5 vs E17.5 (Table 1, Additional file 8). Comparing differentially expressed genes observed in *Pkd1*^*-/- *^at E14.5 vs E17.5 (2287 genes) and in WT at E14.5 vs E17.5 (1189 genes) identified genes that were specifically changing during development in diseased (Additional file 9) or healthy conditions (Additional file 10) as shown in Figure 4. This comparison also indicated genes that were regulated during aging from E14.5 to E17.5 regardless of the genotype (Figure 4; Additional file 11). These results indicate that maturation accounted for the greatest number of changes in gene expression, more so than the cyst formation. Nevertheless, 1397 genes (Additional file 9) could be identified for which changes in gene expression levels were specific for *Pkd1*^*-/- *^kidneys. Thus, this analysis served to identify a set of genes specifically changing in PKD that can yield insights about the regulation of gene expression during cystogenesis.

**Table 1:** Summary of all the analyses

			Mutant vs WT at E14.5	Mutant vs WT at E17.5	E17.5 Mutant vs E14.5 Mutant
1	Differentially expressed genes	Up-regulated in mutant	228	512	1284
		Down-regulated in mutant	226	372	1003

2	GO categories (Biological Processes) enrichment	Up-regulated in mutant	189	362	91
		Down-regulated in mutant	157	271	42

3	a) Curated pathways	Up-regulated in mutant	46	52	31
		Down-regulated in mutant	68	23	22

	b) Enriched TFs	Up-regulated in mutant	31	35	12
		Down-regulated in mutant	29	18	6

### Profiling gene expression changes in Pkd1^-/- ^mutants at E14.5 and E17.5

At E14.5, *Pkd1*^*-/- *^mutants do not exhibit any cyst formation (Figure 2). Therefore gene expression changes at E14.5 may provide an indication of signaling pathways that cause cysts rather than being a consequence of cyst formation. Among the most interesting genes identified by microarrays were P2rx7, Cer1, Frzb, Wnt7b, Dvl3, Gpr34, and Gpr116. These genes are representative of calcium, Wnt and GPCR signaling, and their roles are discussed later.

*Pkd1*^*-/- *^mutant embryos show numerous large renal cysts at E17.5. Microarray analysis showed up-regulation of multiple developmental genes such as Bmp8b, Grem1, Mmp12, Sema3c, Sema4c and Sema6c and transcription factors (TFs)- Gli2, Foxo1, Foxp2, Hoxb8, Pou2f1 and Zbtb32 at E17.5 in *Pkd1*^*-/- *^mutant kidneys compared to WT. Many of these genes are essential for ureteric bud formation, outgrowth, and branching during kidney development. On the other hand, the expression of genes associated with the differentiation of specific nephron segments, such as Umod, Pck1, Fmo2, Slc12a3, Pvalb, and Angpt2 were down-regulated in the null mutants. These data suggest that *Pkd1*^*-/- *^mutants have failed to maintain nephron segment-specific transcriptional signatures. Additionally, we found changes in expression of genes previously related to cyst growth and progression such as components of MAPK and JAK/STAT signaling (these are detailed later in the following sections).

### Gene pathway analysis using GO term enrichment and Gene Set Enrichment Analysis

Gene Ontology (GO) and Gene Set Enrichment Analysis (GSEA; http://www.broad.mit.edu/gsea/) are two bioinformatics approaches to identify pathways involved in specific biological processes such as development and disease. GO term enrichment uses association of Gene Ontology terms to genes in a selected gene list. This method discovers what a set of genes may have in common by examining annotations and finding significant shared GO terms whereas GSEA determines whether a known set of genes shows statistically significant, concordant differences between two biological conditions, e.g. WT and *Pkd1*^*-/-*^.

Table 2 and Additional file 12 show the number of enriched GO terms associated with up- and down-regulated genes for each comparison. Of the 1892 gene sets tested in GSEA, we found that 117 (46 up- and 71 down-regulated) pathways at E14.5 and 75 (52 up- and 23 down-regulated) pathways at E17.5 were dysregulated in the mutants. We defined overrepresented pathways by a nominal (NOM) p-value ≤ 0.05. Table 3 and Additional file 13 show enriched gene sets in mutants for up- and down-regulated genes at E14.5 and E17.5. Some pathways may be represented by multiple independent gene sets.

**Table 2:** GO category enrichment: Biological Processes - overrepresented

Mutant at E14.5	Mutant at E17.5
***Up-regulated***	

Establishment or maintenance of apical/basal cell polarity	Calcium-mediated signaling
Vesicle localization	Programmed cell death
Cytosolic calcium ion transport	Negative regulation of transcription
Calcium ion transport into cytosol	Cell development
Blood vessel development	Cell motion
Vasculature development	Apoptosis
Tube development	Notch signaling pathway
Renal water transport	Cell cycle arrest
cAMP catabolic process	Transforming growth factor beta receptor signaling pathway
Fibroblast growth factor receptor signaling pathway	Regulation of cell-cell adhesion
Renal system process involved in regulation of blood volume	Kidney development
Production of nitric oxide during acute inflammatory response	Cell migration
Extracellular matrix organization	Fluid transport
Vasopressin secretion	Extracellular polysaccharide biosynthetic process
Proteolysis	Wnt receptor signaling pathway
	Cell-cell adhesion mediated by integrin
	Fibroblast growth factor receptor signaling pathway
	

***Down-regulated***	

Wnt receptor signaling pathway, calcium modulating pathway	Positive regulation of cytokine secretion
Cell-cell signaling	Positive regulation of gene expression
G1/S transition of mitotic cell cycle	Positive regulation of MAP kinase activity
Carbon metabolism	Metabolic processes
Glycolysis	Cell motility
Amino acid metabolism	Developmental process
RNA splicing	DNA replication
Positive regulation of cell division	Antigen processing

**Table 3:** Differentially regulated gene sets in *Pkd1*^*-/- *^animals

a) E14.5		
***Up-regulated***		*NES*

HSA04630_JAK_STAT_SIGNALING_PATHWAY	JAK-STAT Pathway	1.34
NGUYEN_KERATO_DN	Notch Signaling	1.32
HSA04920_ADIPOCYTOKINE_SIGNALING_PATHWAY	Adipocytokine Signaling	1.3
HSA04210_APOPTOSIS	Apoptosis	1.13
WNT_TARGETS	Wnt Signaling	1.43
PARP_KO_UP	Genomic Integrity	1.27
RACCYCDPATHWAY	Cell Cycle	1.2
REN_E2F1_TARGETS	Cell Cycle	1.19

***Down-regulated***		

LYSINE_DEGRADATION	Amino Acid Metabolism	-1.76
PROPANOATE_METABOLISM	Carbohydrate Metabolism	-1.64
CROONQUIST_IL6_RAS_DN	JAK-STAT Pathway	-1.44
YU_CMYC_UP	Myc regulated genes	-1.37
HSA00330_ARGININE_AND_PROLINE_METABOLISM	Amino Acid Metabolism	-1.27
HSA00620_PYRUVATE_METABOLISM	Carbohydrate Metabolism	-1.25
GLUCONEOGENESIS	Carbohydrate Metabolism	-1.21
GLYCOLYSIS	Carbohydrate Metabolism	-1.21
CELL_ADHESION_RECEPTOR_ACTIVITY	Cell Adhesion	-1.17
BETA_ALANINE_METABOLISM	Amino Acid Metabolism	-1.17
LEE_MYC_E2F1_DN	Myc regulated genes	-1.16
KERATINOCYTEPATHWAY	Mitogenic Pathway	-1.28
GLYCOGEN_METABOLISM	Carbohydrate Metabolism	-1.26
LI_FETAL_VS_WT_KIDNEY_UP	Wilm's Tumor Signature	-1.19
FATTY_ACID_METABOLISM	Lipid Metabolism	-1.11
		
**b) E17.5**		

***Up-regulated***		

EGF_HDMEC_UP	Growth Factor Signaling	1.76

G1_TO_S_CELL_CYCLE_REACTOME	Cell Cycle	1.43
WNTPATHWAY	Wnt Signaling	1.35
CROONQUIST_IL6_RAS_DN	JAK-STAT Pathway	1.31
HSA04010_MAPK_SIGNALING_PATHWAY	MAPK Signaling	1.23
ZMPSTE24_KO_UP	Ageing	1.15
HSA04520_ADHERENS_JUNCTION	Cell Communication	1.13
P38MAPKPATHWAY	MAPK Signaling	1.02
WILLERT_WNT_NCCIT_ALL_UP	Wnt Signaling	1.43
HIF1_TARGETS	Hypoxia Pathway	1.4

***Down-regulated***		

TRYPTOPHAN_METABOLISM	Amino Acid Metabolism	-1.72
PROPANOATE_METABOLISM	Carbohydrate Metabolism	-1.38
HSA00970_AMINOACYL_TRNA_BIOSYNTHESIS	Translation	-1.23
VALINE_LEUCINE_AND_ISOLEUCINE_DEGRADATION	Amino Acid Metabolism	-1.14
HSA00564_GLYCEROPHOSPHOLIPID_METABOLISM	Lipid Metabolism	-1.18
GLUCONEOGENESIS	Carbohydrate Metabolism	-1.11
GLYCOLYSIS	Carbohydrate Metabolism	-1.11

NES represents the degree of enrichment of the gene set at the top or bottom of the ordered gene list.

NOM P-value measures the significance of NES for a gene set by using permutation testing. NOM P-value < 0.001 is used for table 3.

We found that most of the down-regulated gene sets in *Pkd1*^*-/- *^mutants compared to WT at E14.5 and E17.5 represent metabolic pathways (Table 3 and Additional file 13), as suggested by GSEA and GO analysis. In contrast, both the GSEA and GO analysis of up-regulated gene sets in *Pkd1*^*-/- *^mutants compared to WT at E14.5 and E17.5 suggested that *Pkd1*^*-/- *^mutants displayed a rich gene transcriptional profile for kidney development and regeneration, including Wnt, calcium, MAPK and TGFβ signaling (Additional file 13). Among the pathways identified by GO and GSEA were the following:

#### Activation of mitogenic signaling pathways

We identified 11 up-regulated gene sets in *Pkd1*^*-/- *^mutants compared to WT associated with mitogenic signaling at E17.5, including those associated with growth factor/receptor tyrosine kinase (RTK) signaling (e.g. FGFs, EGF) and extracellular cellular matrix (ECM)/integrin signaling. Previous studies have suggested that activation of EGFs, FGFs and their receptors could promote tubular epithelial cell proliferation and cyst formation in PKD [35-37]. We found Fgfr1 (3.10 fold), Fgfr3 (10.16 fold), Fgf10 (4.18 fold), Nrg1 (3.37 fold) and Prkcb (2.56 fold) up-regulated in *Pkd1*^*-/- *^mutants compared to WT at E17.5. Although gene sets associated with G protein-coupled receptor (GPCR) were not definitively enriched (p = 0.07) in the cystic kidneys at E17.5 (two gene sets associated with GPCR were enriched at E14.5 in *Pkd1*^*-/- *^mutants compared to WT) as shown in Additional file 13, we identified 17 up-regulated individual genes associated with GPCR. Of interest, genes involved in cAMP mediated signaling and calcium regulation such as Pde4b (7.22 fold), Calcr (6.14 fold), and Sstr2 (11.4 fold) were all up-regulated, while negative regulator of GPCR signaling such as Gprasp1 (59.82 fold), and Rgs3 (5.14 fold) [38] were down-regulated. These results suggest changes in GPCR signaling that might be associated with intracellular cAMP and calcium regulation in the kidneys of *Pkd1*^*-/- *^animals.

Increased cAMP has been shown to promote renal cystic epithelial proliferation in PKD [39]. We observed up-regulation of adenylyl cyclase, Adcy7, which may increase cAMP production in the renal cysts, which in turn promotes renal cystic epithelial proliferation in PKD [39]. The change in Ca^2+ ^homeostasis is an important feature of ADPKD and may lead to increased levels of cAMP [39-41]. Although the gene sets for calcium/calcineurin/NFAT signaling were not significantly enriched (p = 0.14), we found that Crebbp (2.08 fold), P2rx7 (11.66 fold), Prkcb (2.55 fold) and Traf2 (2.14 fold) were up-regulated in the *Pkd1*^*-/- *^cystic kidneys, suggesting a situation where limitation of intracellular Ca^2+ ^could promote cAMP mediated activation of B-Raf/MEK/ERK pathway and cellular proliferation [39-41].

Previous studies suggested that aberrant activation of ERK/MAPK signaling might modulate cyst growth in ADPKD. We found three gene sets for ERK/MAPK signaling cascades up-regulated in *Pkd1*^*-/- *^kidneys compared to WT at E17.5 (Table 3 and Additional file 13) including up-regulation of Fgfr3 (10.16-fold), Map3k12 (2.32-fold), Mink1 (23.48-fold), Rps6ka5 (2.14-fold), and Fosb (2.25-fold). Although we found slight up-regulation of Akt1 (1.71-fold) and Eif4e (2.33-fold), no definitive enrichment of the mTOR pathway could be determined. This may be important from a therapeutic perspective: the mTOR pathway has been considered as a drug target in arresting ADPKD progression, despite recent studies in humans with ADPKD that did not demonstrate a therapeutic effect of Sirolimus and Everolimus, inhibitors of the mTOR pathway, on cyst progression [42,43].

#### Activation of angiogenic and immune/inflammatory pathways

Activation of immunoregulatory and inflammatory pathways may be involved in cyst growth in ADPKD. Multiple up-regulated gene sets in the *Pkd1*^*-/- *^kidneys compared to WT at E17.5 were associated with immune/inflammatory (JAK-STAT (n = 3), immunoregulation and inflammation (n = 2)) responses including up-regulation of Il1rl1 (3.79-fold), Il28ra (2.26-fold), Il4i1 (7.35-fold), Jakmip1 (2.6-fold) and Jakmip2 (2.57-fold). We found increased expression of cytokines such as Cxcl1 (2.19-fold), Cxcl16 (2.26-fold), and Clcf1 (8.57-fold). We also found two dysregulated pathways for aging in *Pkd1*^*-/- *^kidneys compared to WT at E17.5 (Additional file 13).

#### Activation of HDAC inhibitor pathways

Recently it has been shown that inhibition of class I HDACs is able to suppress *Pkd2 *related phenotypes [24]. Our studies have not implicated any class I HDACs in the pathogenesis of PKD1. On the other hand, we found down-regulation of a member of the class II HDACs, Hdac9 (22.29-fold) in *Pkd1*^*-/- *^mutants compared to WT at E17.5. Additionally, we found multiple up-regulated gene sets belonging to the class of histone deacetylase inhibitors (n = 7; Additional file 13) in *Pkd1*^*-/- *^kidneys compared to WT at E17.5. HDAC9 plays a role in hematopoiesis, and its deregulated expression, along with altered expression of TGF-β2, may be associated with human cancer and Peters' anomaly [44,45]. As reported in the KEGG pathway database (http://www.genome.jp/kegg/pathway.html), histone deacetylases are involved in many pathways including signal transduction, the notch signaling pathway, cell growth, and death/cell cycle. Their precise role in *Pkd1 *dependent ADPKD remains obscure. However, strong differential regulation of genes involved in the class II HDAC regulatory cascade suggests that histone modification may be an important event in transcriptome reprogramming during PKD progression.

### Comparison with other ADPKD data sets in mouse and human

We aimed to derive correlations between the gene expression changes in PKD and a set of pathways that modulate disease severity in our mouse model and human ADPKD. We compared our study with another published study using the *Pkd1*^*L3/L3 *^mouse model [28] and with the data set available on human ADPKD [29]. Chen et al. described members of three pathways, Wnt, Notch, and BMP, as differentially regulated in the *Pkd1*^*L3/L3 *^model compared to the control WT [28]. Our data from the *Pkd1*^*-/- *^model confirmed the differential regulation of members of these pathways (Additional files 14 and 15). A total of 102 dysregulated genes were common between the *Pkd1*^*L3/L3 *^model data and our set of differentially expressed genes at E17.5 (comparison: *Pkd1*^*-/- *^mutants vs. WT), whereas 36 dysregulated genes were common between our *Pkd1*^*-/- *^model at E14.5 (comparison: *Pkd1*^*-/- *^mutants vs. WT) and the *Pkd1*^*L3/L3 *^model. A ~20% commonality was observed at the pathway level between our *Pkd1*^*-/- *^model and the *Pkd1*^*L3/L3 *^model. The comparison between our data set (differentially expressed genes at E17.5) with human ADPKD data [29] showed a 50% overlap between significantly enriched pathways (Table 4, Additional file 16), which included several important pathways such as calcium signaling, Wnt, MAPK, TGFβ signaling pathways, immune/inflammatory responses, and Notch signaling pathways. On the individual gene level, 20% (n = 314) of genes were similarly changed in both our mouse model and human ADPKD (Table 4, Additional file 16).

**Table 4:** Top 50 differentially regulated genes common between our mouse and human ADPKD data from Song et al. [29]

Down-regulated			Up-regulated		
**Gene name**	**Mouse**	**Human**	**Gene name**	**Mouse**	**Human**

Abcb4	371.51	1.90	Adcy7	2.63	2.38
Acot2	4.81	1.81	Amph	218.83	2.34
Acox2	2.70	1.97	Arhgap10	2.61	2.43
Aldh8a1	2.37	91.29	Asph	3.96	2.84
Apom	2.22	18.96	Bcat1	15.08	4.95
Apoo	4.00	2.45	Camsap1l1	10.96	2.21
Bhmt2	2.26	4.78	Ccdc109b	4.48	3.17
Casc1	10.94	2.84	Cdc42bpa	2.70	1.57
Cldn10	2.25	25.93	Eif3a	98.69	2.14
Cpn2	10.64	2.07	Emilin1	4.06	4.78
Cyp8b1	12.17	30.21	Ezh1	3.45	1.80
Dpep1	7.37	17.20	Fam98b	352.24	1.63
Enpp5	3.11	2.59	Foxp2	26.87	3.75
Enpp6	2.21	8.65	Gli2	4.39	4.29
Fam151a	3.21	6.02	Gramd1a	6.84	1.92
G6pc	11.06	4.88	Grem1	2.98	37.73
Gls	2.35	5.69	Ier2	2.67	3.25
Hgd	2.56	38.57	Jph2	39.24	1.85
Hrg	24.10	16.90	Klhl28	4.94	1.78
Itih2	6.35	2.45	Ltbp1	36.39	5.33
Kctd14	6.15	1.62	Mid1	2.59	2.51
Kng1	2.68	81.30	Nnmt	49.60	1.96
Larp5	7.00	1.57	Odf3b	13.13	1.56
Miox	28.27	20.49	Pcdh7	3.18	11.00
Napepld	2.10	2.65	Pcdh9	40.71	4.90
Napsa	3.60	10.05	Pdcl	75.38	1.71
Odz2	3.97	2.15	Pi4kb	2.90	1.75
Pck1	3.28	58.93	Pknox2	7.28	2.84
Picalm	4.93	1.71	Pml	5.69	1.65
Prap1	3.25	5.24	Pprc1	6.17	1.77
Prlr	2.31	13.73	Rab9b	55.23	2.42
Prodh2	5.34	5.45	Radil	2.92	2.18
Psen1	20.62	2.05	Rpl37	3.29	3.04
Pvalb	2.89	3.59	Sema4c	3.13	1.63
Qpct	2.34	2.20	Sept7	3.12	2.87
Rassf6	10.76	3.30	Setd7	21.09	2.57
Rgs3	5.14	2.01	Sfi1	4.63	1.67
Serpinf2	2.16	2.49	Sfxn3	14.28	2.60
Slc13a2	5.87	7.03	Slitrk5	7.02	14.33
Slc16a4	3.38	4.32	Smurf1	2.90	1.71
Slc26a7	2.82	26.10	St5	2.54	1.62
Slc2a9	4.28	11.81	Syne1	6.02	3.12
Slc4a1	3.62	2.57	Synj2	27.19	3.23
Slc5a10	8.43	11.22	Thg1l	2.91	1.69
Slc8a1	2.11	4.08	Tns1	2.66	1.75
Tbc1d4	4.20	1.66	Vps13b	6.39	1.76
Treh	2.66	2.29	Wfdc3	145.61	1.66
Trim15	235.85	1.97	Yy1	3.48	1.72
Ttr	6.52	1.93	Zbtb10	4.06	1.62
Umod	3.41	210.17	Zfhx4	2.87	4.19

### Transcription factor analysis of Pkd1^-/- ^kidneys

Computational analysis of genetic regulatory regions of co-expressed genes can furnish additional information on the regulation of a gene set by specific transcription factors (TFs) [29]. Using GSEA, we searched for overrepresented TF promoter binding motifs among differentially expressed genes (Table 1). We defined overrepresented TF binding motifs by a NOM p-value ≤ 0.05. Additional file 17 lists all the dysregulated gene sets with shared TF binding sites for renal development, mitogen-mediated proliferation, cell cycle, epithelial-mesenchymal transition, angiogenesis, and immune/inflammatory response. Several of these enriched transcription factors, such as Gli2 (4.39 fold), Foxo1 (2.51 fold) and Pou2F1 (2.88 fold) were also differentially regulated in our microarray analyses, providing additional evidence for their functional relevance in PKD.

### Prediction of miRNA and miRNA-target interactions, and analysis of miRNA expression

	Large scale transcriptional reprogramming during the progression of PKD is consistent with a complex, multi-layered regulatory process, one of the possible regulators of which are miRNAs. Insights into the biological pathways potentially regulated by miRNAs in PKD were obtained by predicting target miRNAs (Additional file 1) using four prediction tools, TargetScan [31], miRanda [32], miRDB [33] and microT [34] for the differentially expressed genes in cystic kidneys at E14.5 and E17.5 (Table 5). Results were overlapped using custom written Perl scripts (Additional file 2). Integrating the results from several prediction algorithms helps in reducing false positives [13]. More than two tools predicted over 5000 mRNA-miRNA interactions, which represented approximately 500 dysregulated genes in cystic kidneys were predicted targets of at least one miRNA. A total of 344 miRNAs were predicted to target 179 out of 454 dysregulated genes at E14.5 by different algorithms (Table 5a and Additional file 18), whereas 372 out of 884 dysregulated genes were predicted targets of 424 miRNAs at E17.5 (Table 5b and Additional file 19). Of these 372 and 179 dysregulated genes at E17.5 and E14.5 respectively, about 82% were predicted targets of multiple miRNAs (Table 5, Additional files 18 and 19), thus suggesting a role of miRNAs in PKD transcriptional reprogramming. These results are consistent with the previous studies estimating that nearly all mammalian genes may be regulated by miRNAs [31].

**Table 5:** Target miRNA prediction for significantly regulated genes at (a) E14.5 and (b) E17.5

(a) E14.5	miRanda	miRDB	TargetScan *1348 miR-gene*	TargetScan and miRDB
microT *1329 miR-gene*	187 miR-gene	52 miR-gene	10 miR-gene	4 miR-gene
miRanda *11350 miR-gene*	-	809 miR-gene	336 miR-gene	104 miR-gene
miRDB *2009 miR-gene*	-	-	137 miR-gene	-
microT and miRanda	-	37 miR-gene	8 miR-gene	4 miR-gene

				

**(b) E17.5**	**miRanda**	**miRDB**	T**argetScan *****3229 miR-gene***	**TargetScan and miRDB**

microT *2478 miR-gene*	504 miR-gene	118 miR-gene	30 miR-gene	4 miR-gene
miRanda *25540 miR-gene*	-	2114 miR-gene	892 miR-gene	269 miR-gene
miRDB *4492 miR-gene*	-	-	331 miR-gene	-
microT and miRanda	-	82 miR-gene	20 miR-gene	4 miR-gene

### Validation of expression by qPCR

**Figure 5:**
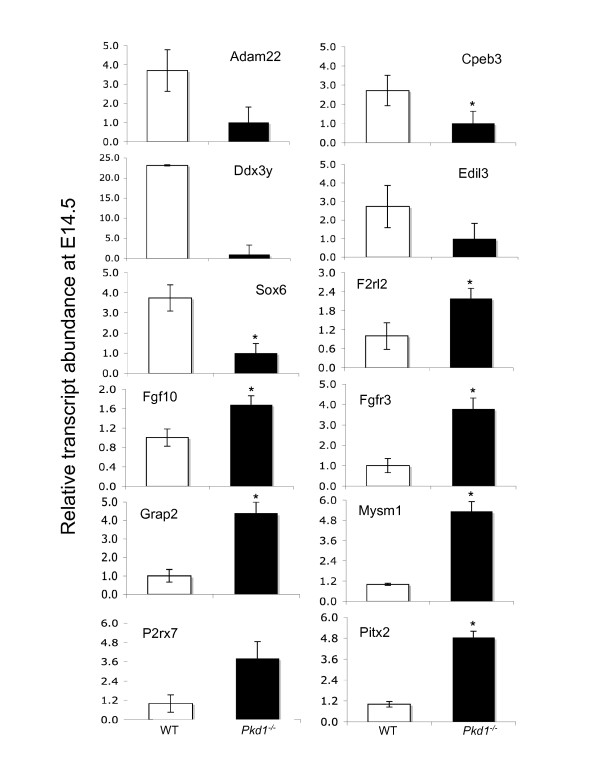
**qPCR analysis of mRNAs in PKD at embryonic age 14**. Expression of 12 genes (Ddx3y, Adam22, Cpeb3, Sox6, Edil3, Mysm1, Pitx2, F2rl2, Grap2, P2rx7, Fgfr3 and Fgf10) significantly regulated on mRNA microarray was verified using qPCR assays. Ddx3y, Adam22, Cpeb3, Sox6 and Edil3 were down-regulated in *Pkd1*^*-/- *^kidneys. Mysm1, Pitx2, F2rl2, Grap2, P2rx7, Fgfr3 and Fgf10 were up-regulated in *Pkd1*^*-/- *^kidneys. '*' significantly different at p-value < 0.05.

**Figure 6:**
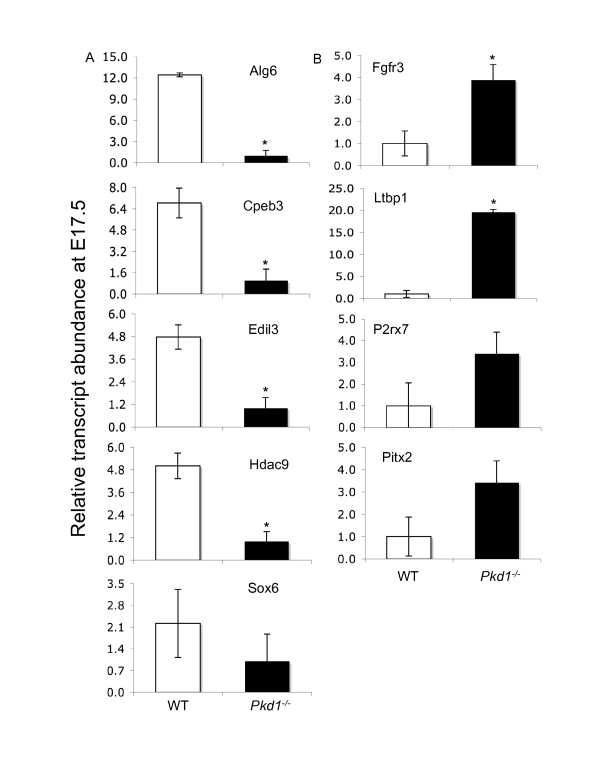
**qPCR analysis of mRNAs in PKD at embryonic age 17**. Expression of 9 genes (Alg6, Cpeb3, Edil3, Hdac9, Sox6, Ltbp1, Fgfr3, P2rx7 and Pitx2) significantly regulated on mRNA microarray was verified using qPCR assays. **(A) **Alg6, Cpeb3, Edil3, Hdac9 and Sox6 were down-regulated in *Pkd1*^*-/- *^kidneys whereas Ltbp1, Fgfr3, P2rx7 and Pitx2 were up-regulated **(B) **in *Pkd1*^*-/- *^kidneys. '*' significantly different at p-value < 0.05.

Using qPCR assays for the *Pkd1*^*-/- *^and WT samples, we confirmed the microarray results on a subset of genes that were- (i) differentially expressed on microarrays, (ii) known/suggested to be involved in ADPKD, and (iii) targets of miRNAs predicted by two or more tools. These genes were P2rx7, Cpeb3, Hdac9, Sox6, Calcr, Pitx2, Fgfr3, Fgf10, Adam22, Ddx3y, F2rl2, Grap2, Edil3, Mysm1 and Alg6 (Figures 5 and 6). Qualitatively, the qPCR validation data agreed with the microarray data (Additional file 20). On the other hand, we found that for certain genes such as Adam22, Grap2 and F2rl2 the fold changes observed on microarrays varied from those obtained by qPCR (Additional file 20). Several factors may be responsible for these variations, among which include the distinct platforms used for microarrays and qPCR, differences in qPCR amplicon/primers and hybridization probes, non-specific and cross-hybridization and issues associated with amplification of mRNA samples [46,47].

**Figure 7:**
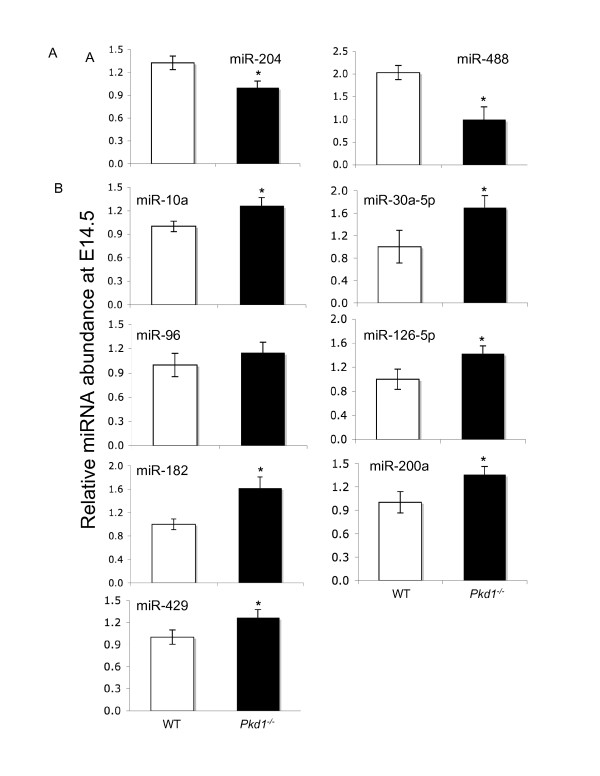
**qPCR analysis of miRNAs in PKD at embryonic age 14**. Expression of 9 miRNAs (miR-204, miR-488, miR10a, miR-30a, miR-96, miR-126-5p, miR-182, miR-200a and miR-429), predicted to target significantly regulated genes at E14.5 was assayed using miRNA-qPCR. miR-204 and miR-488 **(A) **were down-regulated in *Pkd1*^*-/- *^kidneys whereas miR10a, miR-30a, miR-96, miR-126-5p, miR-182, miR-200a and miR-429 **(B) **were up-regulated in *Pkd1*^*-/- *^kidneys. '*' significantly different at p-value < 0.05.

**Figure 8:**
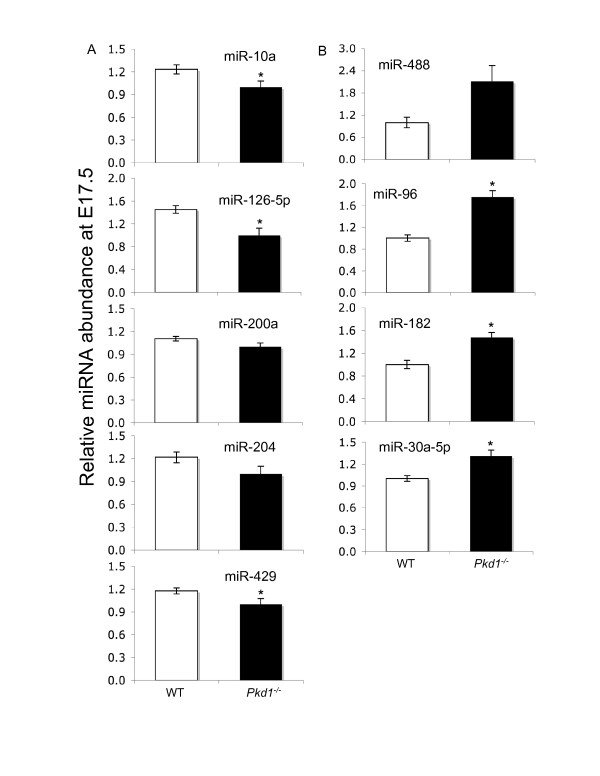
**qPCR analysis of miRNAs in PKD at embryonic age 17**. Expression of 9 miRNAs (miR-10a, miR-126-5p, miR-200a, miR-204, miR-429, miR-488, miR-96, miR-182 and miR-30a-5p), predicted to target significantly regulated genes at E17.5 was evaluated using miRNA-qPCR assays. **(A) **miR-10a, miR-126-5p, miR-200a, miR-204 and miR-429 were down-regulated in *Pkd1*^*-/- *^kidneys. **(B) **miR-488, miR-96, miR-182 and miR-30a-5p were up-regulated in *Pkd1*^*-/- *^kidneys. '*' significantly different at p-value < 0.05.

Computational identification of a large number of miRNA-mRNA target interactions suggested that the expression of miRNAs might also change during the progression of PKD. We tested this hypothesis by determining the differential expression of 9 miRNAs (mmu-miR-10a, mmu-miR-30a-5p, mmu-miR-96, mmu-miR-126-5p, mmu-miR-182, mmu-miR-200a, mmu-miR-204, mmu-miR-429, and mmu-miR-488) between WT and *Pkd1*^*-/- *^genotypes at E14.5 and E17.5 (Figures 7 and 8). These nine miRNAs are expressed in kidney [48], but have not been previously associated with ADPKD. In our computational analysis, two or more tools predicted them as targets for differentially expressed genes at E14.5 and E17.5. The significance of changes in relative expression of miRNAs among the two groups was tested by Student's t-test, and a cutoff of 1.2 fold [49] and P ≤ 0.05 was used to determine the differential miRNA accumulation.

Among the 9 miRNAs tested at E14.5, only two miRNAs, miR-488 and miR-204, were down-regulated in *Pkd1*^*-/- *^kidneys compared to WT, miR-96 did not change, and the remaining 6 miRNAs were up-regulated in *Pkd1*^*-/- *^kidneys compared to WT (Figure 7). In contrast, at E17.5 miR-96, miR-182 and miR-30a were up-regulated in *Pkd1*^*-/- *^kidneys compared to WT. miR-200a did not show significant variation between the WT and *Pkd1*^*-/- *^kidneys at E17.5 (Figure 8). This indicates that miRNA expression also undergoes reprogramming during PKD. Given that renal cystic- and control- kidneys contained a mixture of cell types, the moderate effects we observed in changes in miRNA levels may in reality be far stronger in specific cell/tissue types.

### miRNA-mRNA interactions

Most miRNAs negatively regulate the levels of expression of their targets: therefore, miRNA-mRNA target pair should be inversely correlated for their expression levels i.e. if the expression of a given miRNA is up-regulated, levels of its target genes should be down-regulated and *vice-versa*. Indeed, this was observed for a large number of predicted miRNA:mRNA interactions. At E14.5, expression of 9 miRNAs was inversely related to expression of 46 potential target genes, indicating that these relationships may be functional miRNA-target combinations in ADPKD (Additional file 21). Similarly, 78 genes that were potential targets of 9 miRNAs were inversely related by their expression at E17.5 (Additional file 21). Several of the genes verified with qPCRs (Figures 5,6), and predicted as targets of the miRNAs, showed a reciprocal relationship (Table 6). For example, down-regulation of miR-200a at E17.5 was correlated with up-regulation of Pitx2 in the *Pkd1*^*-/- *^mutants (Additional file 21). Similarly, down-regulation of miR-10a, miR-126-5p, miR-204, and miR-488 at E17.5 were inversely correlated with up-regulation of Ltbp1, Edil3, P2rx7, and Fgfr3 respectively (Additional file 21). Conversely, we found that up-regulation of miR-30a-5p, miR-96 and miR-182 at E17.5, and miR-429 at E14.5 were reciprocally correlated with down-regulation of Cpeb3, Sox6, Hdac9, and Ddx3y respectively in *Pkd1*^*-/- *^mutants (Additional file 21). Moreover, miRs-10a, -30a-5p, -96, -126-5p, -182, -200a, -204, -429, and -488 have not been previously linked to PKD. Thus, our analysis suggests a potential important role for miRNAs in PKD, though we emphasize that these predicted miRNA:mRNA interaction pairs are subject to further experimental validation.

**Table 6:** Selected inverse gene-(predicted)miRNA relations

Genes	Fold Change gene (E14.5) on microarray	Fold Change Gene (E17.5) on microarray	Target miRNA	Fold Change miRNA (E14.5) from qPCR	Fold Change miRNA (E17.5) from qPCR	Prediction Algorithms	Pathways
Adam22	***-133***		miR-30a-5p	***1.70***	1.31	TargetScan, miRanda	Cell-matrix interaction
Calcr	4.63	6.14	miR-200a	1.36	-1.10	TargetScan, miRanda and miRDB; TargetScan, miRanda	Neuroactive ligand-receptor interaction
Calcr	4.63	6.14	miR-10a	1.26	-1.23	TargetScan, miRanda and miRDB; TargetScan, miRanda	Neuroactive ligand-receptor interaction
Cpeb3	***-19.88***	***-6.47***	miR-30a-5p	***1.70***	***1.31***	TargetScan, miRanda	
Cpeb3	***-19.88***	-6.47	miR-200a	***1.36***	-1.10	TargetScan, miRanda	
Ddx3y	-13		miR-429	1.27	-1.17	TargetScan, miRanda and miRDB	RIG-I-like receptor signaling pathway
Edil3	***-4.82***	-6.43	miR-126-5p	***1.43***	-1.45	TargetScan, miRanda and miRDB	Cell adhesion
Edil3	***-4.82***	***-6.43***	miR-182	***1.62***	***1.48***	TargetScan, miRanda and miRDB	Cell adhesion
F2rl2	35.26		miR-126-5p	1.43	-1.45	TargetScan, miRDB	
Fgf10	1.05	4.18	miR-126-5p	1.43	-1.45	TargetScan, miRanda and miRDB; miRDB and miRanda	MAPK signaling
Fgfr3	***4.12***	10.16	miR-488	***-2.03***	2.11	TargetScan, miRanda and miRDB	MAPK signaling
Grap2	157		miR-10a	1.26	-1.23	TargetScan, miRanda	T cell receptor signaling pathway
Hdac9	-1.54	***-22.29***	miR-30a-5p	1.70	***1.31***	TargetScan, miRanda; TargetScan, miRanda and miRDB	Notch signaling
Hdac9	-1.54	-22.29	miR-204	-1.32	-1.22	TargetScan, miRanda; TargetScan, miRanda and miRDB	Notch signaling
Hdac9	-1.54	***-22.29***	miR-182	1.62	***1.48***	TargetScan, miRanda; TargetScan, miRanda and miRDB	Notch signaling
Ltbp1	-368.46	***36.38***	miR-10a	1.26	***-1.23***	TargetScan, miRanda	TGF-Beta signaling
Mysm1	2.85	***3.24***	miR-126-5p	1.43	***-1.45***	TargetScan, miRanda	
P2rx7	***4.65***	***11.66***	miR-204	***-1.32***	***-1.22***	miRDB, miRanda	Calcium Signaling
Pitx2	1.23	***3.91***	miR-200a	1.36	***-1.10***	TargetScan, miRanda and miRDB	TGF-Beta signaling
Sox6	***-1.94***	***-28.68***	miR-96	***1.15***	***1.76***	TargetScan, miRanda and miRDB; TargetScan, miRanda	Negative regulation of transcription
Sox6	***-1.94***	***-28.68***	miR-182	***1.62***	***1.48***	TargetScan, miRanda and miRDB; TargetScan, miRanda	Negative regulation of transcription

**Figure 9:**
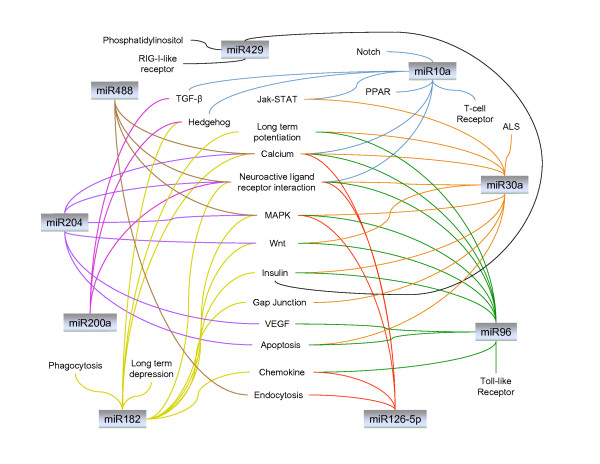
**Overrepresented miRNA regulatory pathways in PKD**. Gene Ontology and Gene Set Enrichment Analysis were used to identify significant enrichment for pathway annotations among differentially expressed target genes of predicted miRNAs in PKD. The figure shows each miRNA targets more than one pathway and each pathway is targeted by more than one miRNA. Only miRNAs validated by qPCR are shown.

We analyzed the functional enrichment of predicted and validated (by qPCR) miRNAs for differentially expressed genes in each comparison in an attempt to uncover the functional meaning among these miRNAs (Figure 9). Genes that were commonly targeted by the differentially expressed miRNAs in *Pkd1*^*-/- *^samples were clustered in 18 biochemical pathways. The overall analysis highlighted that signal transduction pathways such as calcium, VEGF, Notch, and MAPK signaling (Figure 9) may be regulated by miRNA. For example, miR-30a-5p may be involved in histone deactylase inhibitor pathways, apoptosis, calcium and Wnt signaling (Figure 9); miR-10a may be involved in TGF-β and hedgehog signaling; miR-204 may be involved in calcium signaling while miR-488 may be involved in MAPK signaling by targeting Fgfr3 (Figure 9). Thus, we generated a comprehensive atlas of miRNA-target genes and pathways during the progression of PKD. This dataset will serve as a useful resource for future investigations.

## Discussion

**Figure 10:**
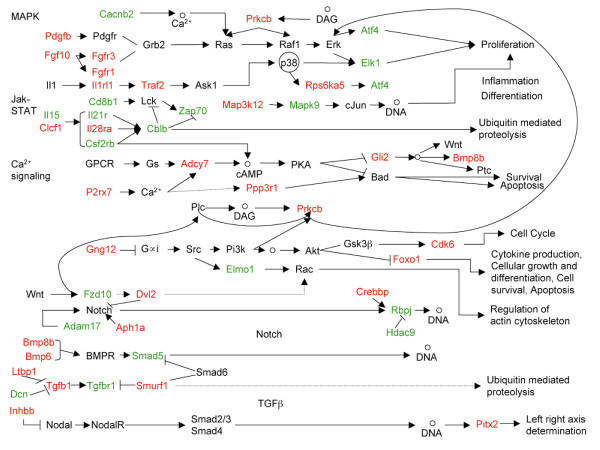
**Schematic summary of dysregulated signalling pathways in *Pkd1*^*-/- *^model**. The figure shows aberrant change in signalling pathways in *Pkd1*^*-/- *^mouse model. Up-regulated genes are shown in red and down-regulated genes in green. Genes that were not differentially expressed are shown in black. Circles designate chemical compounds and DAG stands for diacylglycerol.

In the current investigation, we have attempted to uncover the gene expression signatures associated with the initiation and progression of ADPKD, to decipher the signal transduction pathways associated with these changes in gene expression, and to explore the potential role of miRNAs in effecting these gene expression and signal transduction differences between normal and PKD kidneys. We used a combinatorial approach involving microarrays, data mining and prediction of target miRNAs to profile changes in gene expression during progression of ADPKD, integrate various signaling pathways and present a possible cellular signaling circuitry in PKD (Figure 10). In parallel to the mRNA expression profiling, we also determined the changes in expression of several miRNAs that were computationally identified for their probable roles in ADPKD, thus allowing us to predict several miRNA-mRNA reciprocal interactions. By comparing our datasets with those available for human ADPKD, we found common mis-regulated pathways in the developmental and signaling pathways. Identification of commonly regulated pathways and genes between mice and humans is essential for designing effective therapeutics, as these genes/pathways would be targets for pharmacological intervention in humans with PKD.

### Transcriptome changes in PKD

A high level of complexity in the molecular pathobiology was found in *Pkd1*^*-/- *^cystic kidneys. We identified genes that were changed only in *Pkd1*^*-/- *^animals such as Aqp7, Bmp6, Bmp8b, Ddx3y, Kl, Psen1 and Pitx2. This shows changes in expression of developmental and nephron-segment specific genes. Further we identified changes in expression of genes that may be associated with pre-cystic stages leading to cyst formation such as changes in the components of Wnt signaling. We identified changes in gene expression related to cyst growth such as components of MAPK (e.g. Map3k12, Fgf10, Prkcb, Traf2) and TGF-β (e.g. Pitx2) signaling that were up-regulated in the *Pkd1*^*-/- *^animals. The GO term enrichment and GSEA revealed changes in calcium, MAPK, Wnt, TGF-β and JAK/STAT signaling pathways in the *Pkd1*^*-/- *^animals. Additionally, this analysis showed that the class II HDAC, Hdac9 was strongly down-regulated in *Pkd1*^*-/- *^animals. This could effect more global changes in gene expression.

Mitogen-Activated Protein Kinases play central roles in the signal transduction pathways that affect gene expression, cell proliferation, differentiation and apoptosis [50]. Although some studies have suggested that cellular proliferation may be involved in initial cyst formation in PKD, other evidence suggests that abnormal proliferation contributes to cyst growth rather than cyst initiation [51-54]. Our data (Table 3 and Additional file 6), showing a greater increase in MAPK-associated pathways at E17.5 as compared with E14.5, are more consistent with the latter hypothesis that proliferation is more important in cyst growth than in initiation.

### Wnt signaling in ADPKD

Previous studies have shown that the activation of canonical Wnt signaling [55] and inhibition of non-canonical Wnt signaling (such as decreased activation of c-jun N-terminal kinase, JNK, and transient changes in intracellular Ca^2+ ^concentrations) may play roles in cyst formation in PKD [56]. We found Wnt7b (1.6 fold), Fzd6 (1.3 fold), Dvl3 (1.6 fold) and Lef1 (1.7 fold) were up-regulated while Apc (2.4 fold), an inhibitor of canonical Wnt signaling was down-regulated at E14.5. Although microarray probes failed to detect increased Wnt7a expression, our independent study confirms up-regulation of Wnt7a in *Pkd1*^*-/- *^animals (Qin et al, submitted). Moreover, in a study of *Pkd1*^*L3/L3 *^mouse model Wnt7a and Wnt7b were also found up-regulated in mutant kidneys [28]. The GSEA results also showed enrichment of canonical Wnt signaling for up-regulated genes in *Pkd1*^*-/- *^mutants at E14.5 (Table 3). Canonical Wnt signaling was also up-regulated at E17.5, as determined by both GO analysis and GSEA (Tables 2 and 3, Additional files 12 and 13). Together, these results suggested that there may be an increase in canonical Wnt signaling in *Pkd1*^*-/- *^animals. Additionally, the GO term enrichment analysis showed down-regulation of non-canonical Wnt signaling in *Pkd1*^*-/- *^animals at E14.5 (Table 2, Additional file 12).

In contradistinction to the GO and GSEA analyses suggesting increased Wnt signaling in *Pkd1*^*-/- *^kidneys, we found that two potential inhibitors of Wnt signaling, Cer1 and Frzb (respectively increased 12 fold and 9.7 fold at E14.5), were up-regulated in *Pkd1*^*-/- *^animals at E14.5. Cer1 and Frzb belong to the family of sFRPs (secreted Frizzled Related Proteins) that act as inhibitors of Wnt signaling in animals such as *Xenopus *and chick by interfering with the binding of Wnt proteins to Frizzled transmembrane receptors [57]. Frzb is a known Wnt antagonist in mammals [57,58]. However the mouse Cer1 may not inhibit Wnt signaling [59]. At present we are unable to explain the discrepancy between the overall informatic result that canonical Wnt signaling is increased in *Pkd1*^*-/- *^kidneys, and the observed increased expression of Wnt antagonists Cer1 and Frzb. It is possible that Cer1 and/or Frzb have a greater ability to interfere with non-canonical vs. canonical Wnt signaling, and that this will be borne out by future studies.

### Calcium signaling in ADPKD

The polycystin-1/2 complex (PC1/PC2) mediates Ca^2+ ^entry into the cell in response to mechanical stimulation of the primary cilium [60]. This in turn triggers additional release of Ca^2+ ^from intracellular stores. Thus, disruption of the polycystin pathway alters intracellular Ca^2+ ^homeostasis [1,56]. It has been suggested that altered intracellular Ca^2+ ^levels increases cAMP levels by stimulating adenylyl cyclases [60]. In turn, increased cAMP paradoxically stimulates MAPK/ERK signaling and promotes renal cystic epithelial proliferation in *Pkd1*^*-/- *^cells [39]. Our results showed that in *Pkd1*^*-/- *^animals at E17.5 an adenylyl cyclase, Adcy7 was up-regulated. Thus, increased gene expression of an adenylyl cyclase may be an additional contributing factor to increased cAMP levels in ADPKD.

### Novel regulatory mechanisms in ADPKD

Our study identifies several new nodes of regulation in ADPKD. For example, we found a strong down-regulation of Alg6 (asparagine-linked glycosylation 6 homolog) in the *Pkd1*^*-/- *^genotype at E17.5. ALGs are important components of the glycosylation pathway involving the endoplasmic reticulum (ER) and the Golgi apparatus. Alg6 encodes a member of the glucosyltransferase family that catalyzes the addition of the first glucose residue to the growing lipid-linked oligosaccharide precursor of N-linked glycosylation. Mutations in this gene are associated with congenital disorders of glycosylation type Ic [61]. Indeed, in *Pkd1*^*-/- *^mice, defective glycosylation of protein has been observed, that may have important implications for ADPKD [62,63]. As Alg6 encodes an ER glucosyltransferase [64], its specific downregulation in the *Pkd1*^*-/- *^genotype indicates metabolic reprogramming in the ER and Golgi complex. Thus, exploring Alg6 function in knock-down studies in kidneys may provide new insights regarding the disease processes underlying ADPKD.

The comparison of the *Pkd1*^*-/-*^mouse model and human ADPKD data sets revealed significant overlaps. Some representative of commonly up-regulated genes include Adcy7 (Calcium signaling), Crebbp (Wnt, Jak-STAT, calcium, TGFβ signaling), Cxcl1 (Jak-STAT signaling), Vegfb (VEGF signaling), Dvl2 (Wnt signaling), Tgif1 and Smurf1 (TGFβ signaling). Representative down-regulated genes that were commonly changed between the two data sets include Umod (loop of Henle marker), Rgs3 (GPCR signaling), Pvalb (distal tubule marker), Pck1 (proximal tubule marker), and Lamc3 (Focal adhesion). Thus, these genes may be used as prognostic markers for ADPKD.

### Possible regulatory roles of miRNAs in ADPKD

We examined the possible involvement of miRNAs in ADPKD in the *Pkd1*^*-/- *^mouse model. While several miRNAs can target a single transcript, each miRNA can also have multiple targets [13,31]. Thus, moderate alterations in miRNA levels can have profound effects on the accumulation of their targets [65,66]. However, their role in ADPKD, both at the individual as well as the systems levels, is still under investigation, as only three studies have directly demonstrated changes in miRNA expression in ADPKD [18,22,23]. We have addressed this problem by systemically estimating the possible miRNAs that may target genes deregulated during disease progression (Additional file 1). We have predicted and verified (by qRT-PCR) reciprocal expression of several miRNAs and their predicted targets in *Pkd1*^*-/- *^animals (Table 6). We observed that miRNAs: miRs-10a, -30a-5p, -96, -126-5p, -182, -200a, -204, -429, and -488; and the miRNA-mRNA interactions such as miR-126-5p-Fgf10, miR-488-Fgfr3, miR-182-Hdac9, miR-204-P2rx7 and miR-96-Sox6 (as shown in Table 6) have not been previously reported in ADPKD.

Signaling pathways are ideal candidates for miRNA-mediated regulation, as their components require finely tuned temporal changes in their expression [65,67]. miRNAs may affect the responsiveness of cells to signaling molecules such as TGFβ, Wnt, Notch, and epidermal growth factor [67]. Once a miRNA targets an inhibitor of a signaling cascade, it serves as a positive regulator by either amplifying signal strength or duration, or empowering cell responsiveness to otherwise sub-threshold stimuli. For example, miR-126 promotes angiogenesis and vascular integrity [68] by inhibiting the production of natural repressor (SPRED1 and PIK3R2) of VEGF signaling, suggesting that it may serve as an effective target for anti-angiogenic therapies. We found that miR-126-5p may be involved in calcium, EGF, MAPK signaling, and neuroactive ligand-receptor interaction (Figure 9; Additional file 21). Previous studies [13,65,67] have shown that a single miRNA can act simultaneously on multiple signaling pathways to coordinate their biological effects and concurrently several miRNAs may regulate single pathway (Figure 9). Moreover, the predicted miRNA:mRNA interactions suggested that the involvement of miRNAs in the signaling pathways through their target mRNAs may lead to the dysregulation of these pathways. For example, the interaction of miR-204:P2rx7 (Figure 10) suggested a possibility that the down-regulation of miR-204 may lead to up-regulation of its target gene, P2rx7 at E17.5 in *Pkd1*^*-/- *^kidneys. P2rx7 is a cell-surface, ligand-gated cation channel and its stimulation leads to alteration in the intracellular Ca^2+ ^levels that may further result into dysregulation of calcium signaling. Therefore, by targeting P2rx7, miR-204 may disrupt calcium signaling. Similarly, at E17.5 in *Pkd1*^*-/- *^animals, the up-regulation of Fgfr3 and Fgf10 (components of MAPK signaling) and down-regulation of their target miRNAs- miR-488 and miR-126-5p respectively, may stimulate MAPK signaling and cell proliferation in *Pkd1*^*-/- *^samples.

The functional correlation between the differentially expressed mRNAs and miRNAs as a module in ADPKD revealed a tight post-transcriptional regulatory network at the mRNA level whose alteration might contribute to increased immune response, by either direct miRNA targeting or through secondary proteins. Further progress in the understanding of miRNA activity, the identification of miRNA signatures in different states, and the advancement of miRNA manipulation techniques will be valuable for deciphering the roles of individual miRNAs in PKD. The overall discovery of differentially regulated miRNAs in the different diseases is expected not only to broaden our biological understanding of these diseases, but more importantly, to identify candidate miRNAs as potential targets for future clinical applications.

## Conclusion

We have attempted to reveal the molecular players of PKD in a *Pkd1*^*-/- *^mouse model. Taken together, our study suggests the presence of complex layers of regulation in ADPKD. Our microarray data revealed genes that specifically changed during disease condition. Further we identified genes related to pre-cystic stages and cyst progression. Our model suggests a cascade of signaling events involving up-regulation of canonical- and down-regulation of non-canonical- Wnt signaling that may result in decreased intracellular Ca^2+ ^concentration, resulting in an increase of intracellular cAMP levels that in turn stimulates MAPK/ERK signaling leading to proliferation, followed by increased Jak-STAT signaling and inflammation (Figure 10), ultimately leading to renal failure. Further, we determined gene expression signatures common between the *Pkd1*^*-/- *^mouse model and human ADPKD. These could be used as prognostic markers of disease progression in PKD. Moreover, we add various new components including Alg6, Hdac9 and several miRNAs to the regulatory layers of ADPKD. We predicted that several of the differentially regulated genes are miRNA targets and miRs-10a, -30a-5p, -96, -126-5p, -182, -200a, -204, -429, and -488 may be important players in cellular signalling events leading to PKD. Furthermore, it is interesting to note that these miRNAs have not been previously reported in PKD. It has been proposed that a single miRNA can target more than hundred genes and one gene can be the target of several miRNAs [69,70]. A future challenge will be to systemically identify all of the miRNAs affecting, and regulated by, the dysregulated cell signalling pathways in ADPKD. Extensive functional analyses of these miRNAs and their target genes by performing knockout and over-expression studies, individually and in combination, are likely to open up new avenues for PKD research.

## Authors' contributions

PP designed and carried out the study, analysed and interpreted the data and drafted the manuscript. SQ participated in the experimental validation of the data. JH helped in animal experiments. JAK helped in designing the study, and participated in drafting the manuscript. All authors have given final approval of the version to be published.

## Supplementary Material

Additional file 1 **Schematic representation of target miRNA prediction for significantly regulated genes**. Step-wise approaches used to predict target miRNAs for the significantly regulated genes obtained at three comparisons, shown in Additional file 1. We used four prediction tools- TargetScan, miRanda, miRDB and microT and the results obtained from individual tool were overlapped by custom written Perl scripts. Only those miRNA-mRNA pairs were considered for further study that were predicted by atleast two tools. Click here for file

Additional file 2 **Perl script used for integrating prediction tools results**. Perl script used for integrating prediction tools results Click here for file

Additional file 3 **Gene-primers used for qPCR**. This table shows list of primers used for qPCR analysis of genes Click here for file

Additional file 4 **Design of experiment**. This figure shows the experimental design implied in this study. It shows four comparisons including two stages-E14.5 and E17.5 and two genotypes- *Pkd1*^*-/- *^and wild-type. Click here for file

Additional file 5 **Significantly regulated genes at E14.5**. This table shows the significantly regulated genes obtained for mutant versus wild-type comparison at E14.5 Click here for file

Additional file 6 **Significantly regulated genes at E17.5**. This table shows the significantly regulated genes obtained for mutant versus wild-type comparison at E17.5 Click here for file

Additional file 7 **Significantly regulated genes in wild-types across time points E14.5 to E17.5**. This table shows the significantly regulated genes obtained for wild-types when compared at E14.5 and E17.5 Click here for file

Additional file 8 **Significantly regulated genes in mutants across time points E14.5 to E17.5**. This table shows the significantly regulated genes obtained for mutant when compared at E14.5 and E17.5 Click here for file

Additional file 9 **Significantly regulated genes specific for disease transition**. This table shows the significantly regulated genes that are specific for transition under disease condition. Click here for file

Additional file 10 **Significantly regulated genes specific for healthy transition**. This table shows the significantly regulated genes that are specific for transition under healthy condition. Click here for file

Additional file 11 **Significantly regulated genes specific to aging**. This table shows the significantly regulated genes that are specific for aging and common to both diseased and healthy conditions. Click here for file

Additional file 12 **Gene Ontology terms- overrepresented biological processes**. This table shows the overrepresented Biological Processes from Gene ontology for significantly regulated genes. Sheets 1 through 3 show overrepresented biological processes for up- and down-regulated genes obtained for E14.5 mutant versus wild-type comparison, E17.5 mutant versus wild-type, and mutants at E14.5 versus E17.5. Click here for file

Additional file 13 **Gene Set Enrichment Analysis- overrepresented gene sets or pathways**. This table shows the overrepresented gene-sets/pathways obtained from Gene Set Enrichment Analysis for significantly regulated genes. Sheets 1 through 3 show overrepresented gene-sets for up- and down-regulated genes obtained for E14.5 mutant versus wild-type comparison, E17.5 mutant versus wild-type, and mutants at E14.5 versus E17.5. Click here for file

Additional file 14 **Common genes obtained between *Pkd1*^*L3/L3 *^and *Pkd1*^*-/- *^mouse models**. This figure shows some of the early and late responsive genes we obtained in comparison between *Pkd1*^*L3/L3 *^and *Pkd1*^*-/- *^mouse models. Click here for file

Additional file 15 **Common genes in *Pkd1*^*L3/L3 *^and *Pkd1*^*-/- *^mouse models**. This table shows the common genes and pathways obtained from comparing the *Pkd1*^*L3/L3 *^and *Pkd1*^*-/- *^mouse models. Click here for file

Additional file 16 **Common genes in *Pkd1*^*-/- *^mouse model and human ADPKD**. This table shows the common genes and pathways obtained from comparing the *Pkd1*^*-/- *^mouse model and human ADPKD. Click here for file

Additional file 17 **Gene Set Enrichment Analysis- overrepresented gene sets with shared Transcription Factors**. This table shows the overrepresented overrepresented gene sets with shared Transcription Factors obtained from Gene Set Enrichment Analysis for significantly regulated genes. Sheets 1 through 3 show overrepresented gene-sets with shared Transcription Factors for up- and down-regulated genes obtained for E14.5 mutant versus wild-type comparison, E17.5 mutant versus wild-type, and mutants at E14.5 versus E17.5. Click here for file

Additional file 18 **Predicted target miRNAs for significantly regulated genes at E14.5**. This table shows the predicted target miRNAs for significantly regulated genes at E14.5. Four tools-TargetScan, miRanda, miRDB and microT were used for prediction and results were overlapped. Only those miRNA:gene pairs are shown that are predicted by atleast two tools. Click here for file

Additional file 19 **Predicted target miRNAs for significantly regulated genes at E17.5**. This table shows the predicted target miRNAs for significantly regulated genes at E17.5. Four tools-TargetScan, miRanda, miRDB and microT were used for prediction and results were overlapped. Only those miRNA:gene pairs are shown that are predicted by atleast two tools. Click here for file

Additional file 20 **Comparison of microarrays and qPCR results**. This table shows the fold change obtained from both microarrays and qPCR for significantly regulated genes (that were validated by qPCR). Click here for file

Additional file 21 **miRNA:target inverse relationship**. This table shows the predicted miRNA:mRNA inverse relationship for significantly regulated genes and 9 selected miRNAs. The expression of these nine miRNAs was validated by qPCR. Only those miRNA:mRNA pairs are shown that are predicted by atleast two tools and show inverse correlation to each other. Click here for file
