# Supplementary figures and images for: Systems biology approach to identify transcriptome reprogramming and candidate microRNA targets during the progression of polycystic kidney disease
Source: BMC Syst Biol. 2011 Apr 25;5:56. doi: 10.1186/1752-0509-5-56 (PMC3111376; doi:10.1186/1752-0509-5-56)

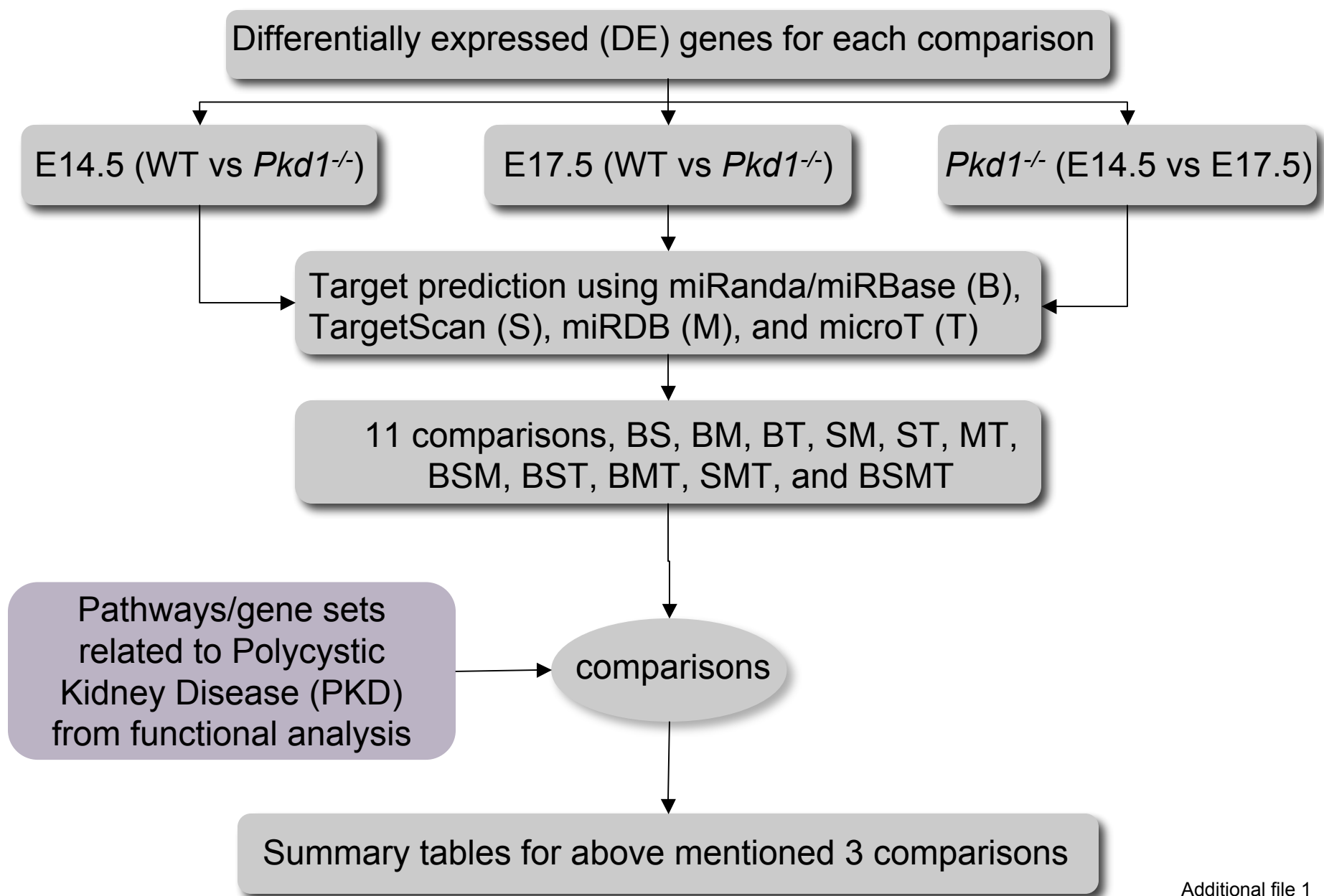

Supplement: Additional file 1 — Schematic representation of target miRNA prediction for significantly regulated genes. Step-wise approaches used to predict target miRNAs for the significantly regulated genes obtained at three comparisons, shown in Additional file 1. We used four prediction tools- TargetScan, miRanda, miRDB and microT and the results obtained from individual tool were overlapped by custom written Perl scripts. Only those miRNA-mRNA pairs were considered for further study that were predicted by atleast two tools. [file 1752-0509-5-56-S1.PDF]

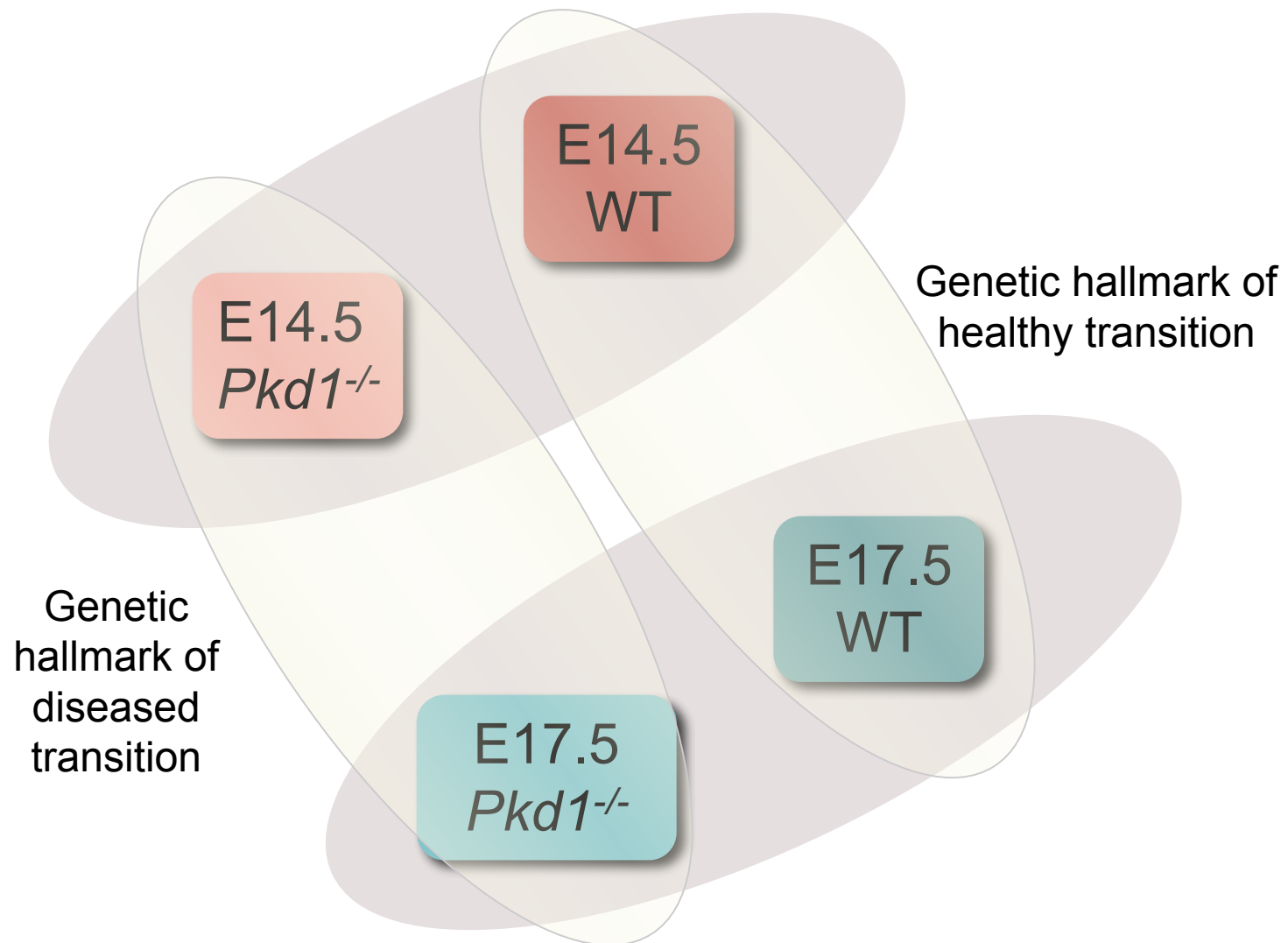

Supplement: Additional file 4 — Design of experiment. This figure shows the experimental design implied in this study. It shows four comparisons including two stages-E14.5 and E17.5 and two genotypes- Pkd1-/- and wild-type. [file 1752-0509-5-56-S4.PDF]

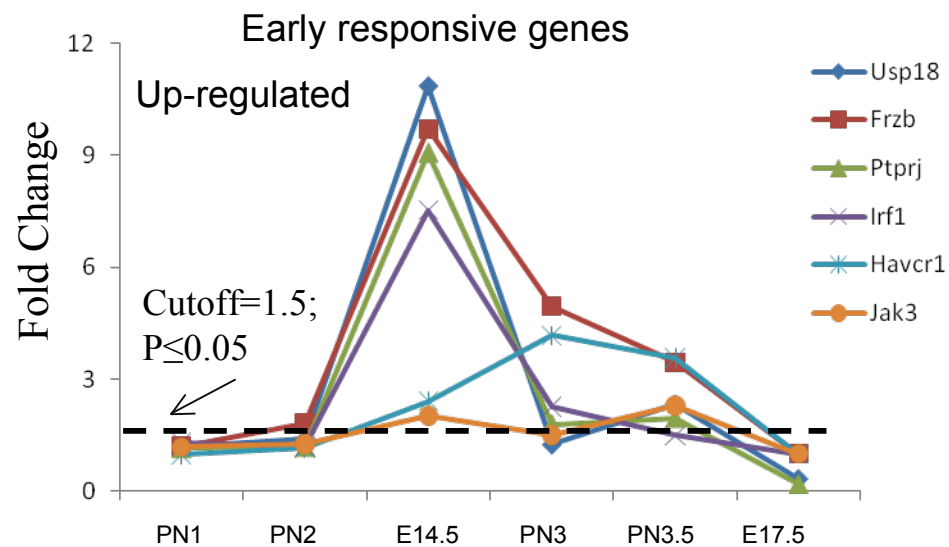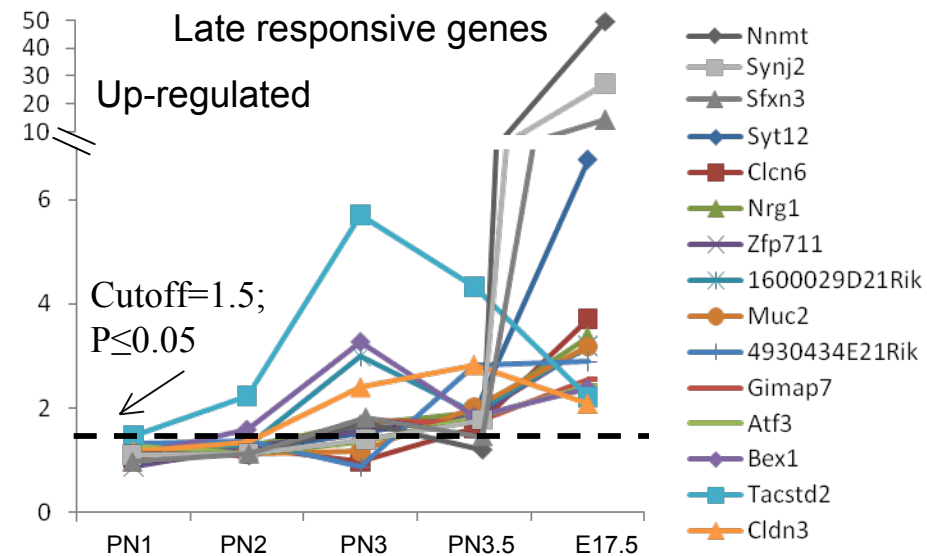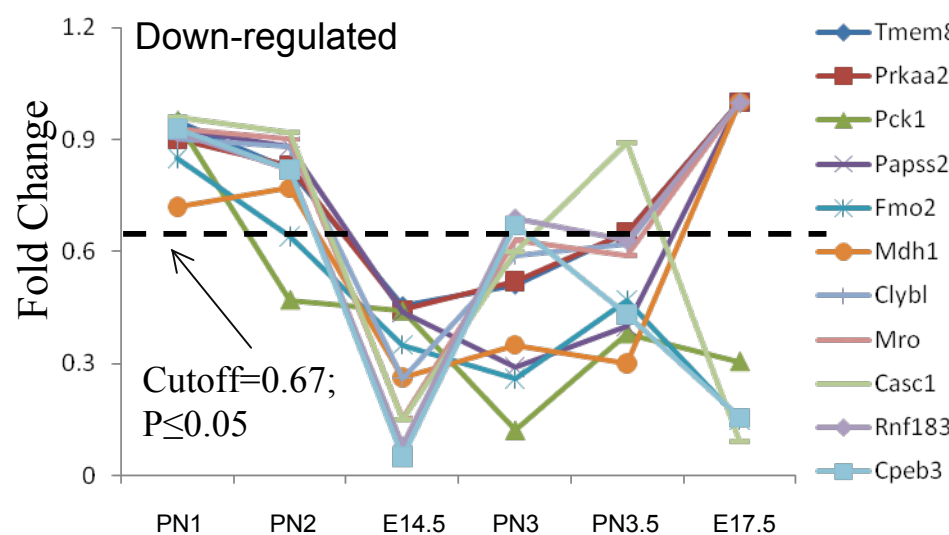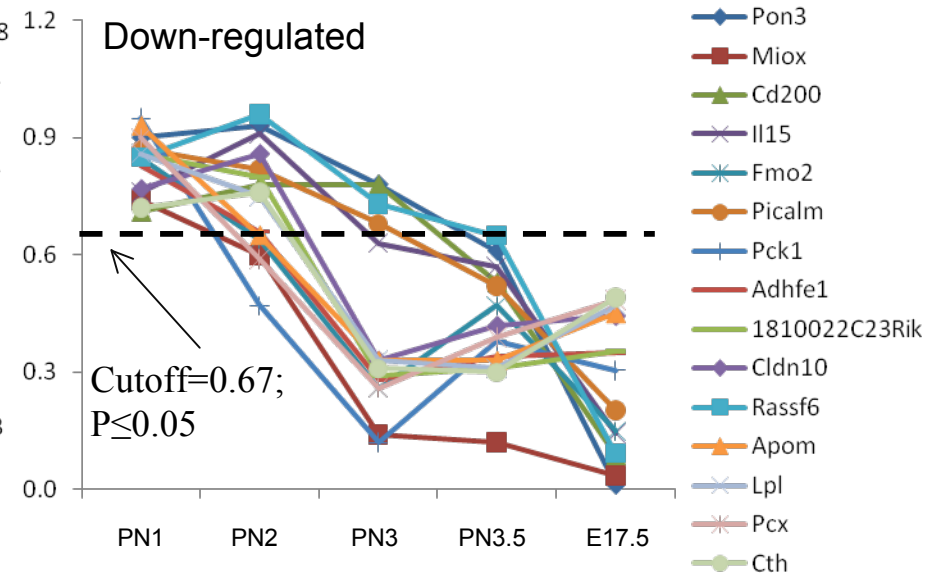

Supplement: Additional file 14 — Common genes obtained between Pkd1L3/L3 and Pkd1-/- mouse models. This figure shows some of the early and late responsive genes we obtained in comparison between Pkd1L3/L3 and Pkd1-/- mouse models. [file 1752-0509-5-56-S14.PDF]
