# Supplementary material for: Systems biology approach to identify transcriptome reprogramming and candidate microRNA targets during the progression of polycystic kidney disease
Source: BMC Syst Biol. 2011 Apr 25;5:56. doi: 10.1186/1752-0509-5-56 (PMC3111376; doi:10.1186/1752-0509-5-56)
Supplement: Additional file 2 — Perl script used for integrating prediction tools results. Perl script used for integrating prediction tools results [file 1752-0509-5-56-S2.PDF]

Additional file 2:
Comparing all the prediction results obtained from the four tools- TargetScan, miRanda, microT and miRDB; taking two files at a time. Similarly the overlapped results were again compared with the remaining result files and there were 11 comparisons performed using the above mentioned 4 tools. Following are the codes for comparing given two files (A) and then removing the duplicate results (B) from the intersection.

[A] Comparing files

```
#!/usr/bin/perl

$firstfile = shift; # input the first prediction result file
$secondfile = shift; # input the second prediction result file

# open datasource
open FIRST, "$firstfile" or die "could not open $firstfile: $!\n";
open SECOND, "$secondfile" or die "could not open $secondfile: $!\n";
open(OUT, ">first_second_overlapped.txt")||die "cannot create
>first_second_overlapped.txt: $!";

$s = 0;

# reading each line of first file
while (<FIRST>) {
$s++;                    # if the first row is the header row
next if ($s == 1);
chomp;
@l = split /\t/;

# splitting each line at the tab and reading each value into variables

        $var1="";

        $var2="";

        $var3="";

        $var4="";



        $var1 = $l[0] if ($l[0]=~/\S+/); # miRNA name

        $var2 = $l[1] if ($l[1]=~/\S+/); # target gene

        $var3 = $l[2] if ($l[2]=~/\S+/); # gene detail
```

```
                $var4 = $l[3] if ($l[3]=~/\S+/); # score or any other relevant information


# similarly opening the second prediction result file
open SECOND, "$secondfile" or die "could not open $secondfile: $!\n";
$s1=0;
while(<SECOND>) { # reading each line of second file one at a time
$s1++;    # if the first row is the header row
next if ($s1 == 1);
chomp;
@l1 = split /\t/; #splitting each line at the tab and reading individual values into variables

                $vr1="";

                $vr2="";

                $vr1 = $l1[0] if ($l1[0]=~/\S+/); # target gene

                $vr2 = $l1[1] if ($l1[1]=~/\S+/); # miRNA name

#comparing two files for the same miRNA targeting the same gene
if (($var1 =~ /$vr2/) && ($var2 eq $vr1))
        {
        # printing the matched miRNA and their target genes with other required
information
        print OUT "$var1\t$var2\t$var3\t$var4\t$vr1\t$vr2\n";
        }

}#close of inner while loop   SECOND

} #close of outer while loop FIRST

close FIRST;
close(OUT)||die "can't close >first_second_overlapped.txt: $!";
```

[B] Removing duplicates

```
#!/usr/bin/perl

$infile = shift; # inputing the “first_second_overlapped.txt” or any of the 11 overlapped
                        #files

open IN, "$infile" or die "could not open $infile: $!\n";
open(OUT, ">unique_first_second_overlapped_genes.txt")||die "cannot create
unique_gene.txt: $!";
```

```
%hash1;
%hash2;
%hash3;
%hash4;
%hash5;

$s=0;
while (<IN>) {
$s++;  # if the first row is the header row
next if ($s == 1);
chomp;

@l = split /\t/;

$sequence ="";
$name="";
$category="";
$var4="";

# reading each column’s values into separate variables

$gene = $l[0] if ($l[0]=~/\S+/);
$name = $l[1] if ($l[1]=~/\S+/);
$gene_detail = $l[2] if ($l[2]=~/\S+/);
$var4=$l[3] if ($l[3]=~/\S+/); # any additional information obtained from prediction tools

# making unique list of miRNAs and their target genes, using miRNA name as unique
if(!defined $hash1{$name}){
$hash1{$name}=$name;
$hash2{$name}=$gene;
$hash3{$name}=$var4;
}else{
$hash2{$name}=$hash2{$name}.";".$gene;
}

}

foreach $id (sort keys %hash1){
print OUT "$hash1{$id}\t$hash2{$id}\t$hash3{$id}\n";
}

close IN;
close(OUT)||die "can't close unique_first_second_overlapped_genes.txt: $!";
```
